# The genus *Myrmarachne* (Araneae, Salticidae) in Flores, Indonesia

**DOI:** 10.3897/zookeys.299.4970

**Published:** 2013-05-14

**Authors:** Takeshi Yamasaki, G. B. Edwards

**Affiliations:** 1 Graduate School of Science and Engineering, Kagoshima University, Kagoshima, 890-0065 Japan; 2 Florida State Collection of Arthropods, FDACS/Division of Plant Industry, P.O.Box 147100, Gainesville, FL 32614-7100, USA

**Keywords:** New species, ant-mimicking jumping spiders, Java, Malay Archipelago

## Abstract

Two new species of the genus *Myrmarachne* are described (*Myrmarachne acutidens* **sp. n.**, *Myrmarachne epigealis* **sp. n.**), and *Myrmarachne macrognatha* and *Myrmarachne melanocephala* are redescribed from Flores specimens. The females of *Myrmarachne macrognatha* are recorded for the first time.

## Introduction

The genus *Myrmarachne* MacLeay, 1839 is one of the largest groups in the family Salticidae, including around 250 species (Prószyński 2012; Platnick 2012). The members of the genus generally resemble ants morphologically and behaviorally, and the resemblance is considered to be due to Batesian mimicry (Wanless 1978). The area from Southeast Asia to Australia through the Malay Archipelago harbors many *Myrmarachne* species. Wallacea, the non-continental transition zone between the Oriental (Sundan) and Australian (Sahulian) zoogeographical regions, is bounded to the west by Wallace’s line and to the east by Lydekker’s line (Hill 2010). The *Myrmarachne* faunas of Wallacean islands have not been studied and revealed sufficiently although Thorell (1877) and Yamasaki (2012) together record ten species from Sulawesi.

Flores forms part of a series of active volcanoes between Java and Timor, and its climate is relatively dry, strongly affected by monsoon and trade winds (Monk et al. 1997; van der Geer 2010). Until now, no *Myrmarachne* species have been recorded from the island. In the present study, we describe two new species and redescribe two species known elsewhere, reported from Flores for the first time.

## Materials and methods

**Figure 1.**
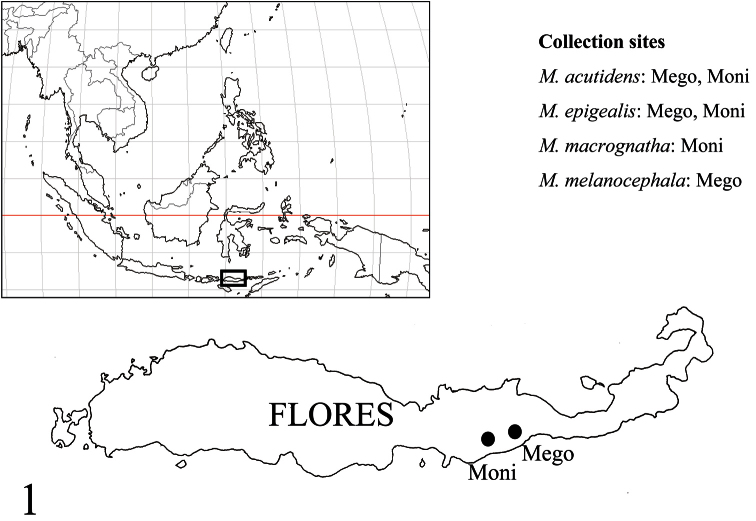
Collection sites. *Myrmarachne acutidens* and *Myrmarachne epigealis* were collected from Mego and Moni, *Myrmarachne macrognatha* from Moni, and *Myrmarachne melanocephala* from Mego.

Collection sites are shown in Figure 1. Morphological observations were made with a Nikon SMZ 1000 stereoscope. Multi-focused montage images were produced using Helicon Focus 4.75 Pro from a series of source images taken by Cannon EOS Kiss x 4 digital camera attached to a Nikon ECLIPSE E600 microscope. Measured parts of the carapace follow Yamasaki (2010). Chelicera length was measured in lateral view, and only for males.

All measurements are given in millimeters. The ranges from the minimum to maximum are shown when more than two specimens were measured. For some species, figures for the holotypes are shown in parentheses. Abbreviations used in the present paper are as follows: AME, anterior median eye; ALE, anterior lateral eye; PME, posterior median eye; PLE, posterior lateral eye; pd, prodorsal; pv, proventral; rd, retrodorsal; RTA, retrolateral tibial apophysis of palp; rv, retroventral.

The type material examined in the present study was borrowed from Naturhistoriska riksmuseet, Stockholm, Sweden (NRM). The type specimens designated here are deposited at Museum Zoologicum Bogoriense, Research Center for Biology, Indonesian Institute of Science, Cibinong, Indonesia (MZB), and at the Florida State Collection of Arthropods, Gainesville, Florida, USA (FSCA).

## Taxonomy

### Myrmarachne acutidens sp. n.

urn:lsid:zoobank.org:act:E48ED428-C691-4C0E-B862-A6E9E0D8E9CD

http://species-id.net/wiki/Myrmarachne_acutidens

Figs 2 –19

#### Type material.

Holotype male (MZB. Aran. 503), Mego [=8°40'S, 122°2'E], Sikka, Flores, East Nusa Tenggara Prov., INDONESIA, 16.X.2012, T. Yamasaki leg. Paratypes: 1 male and 2 females (1 male and 1 female, FCSA; 1 female, MZB. Aran. 504), same data as holotype; 1 male and 1 female (FSCA), Moni [=8°45'S, 121°51'E], Flores, East Nusa Tenggara Prov., INDONESIA, 17.X.2012, T. Yamasaki leg.

#### Diagnosis.

**Figures 2–9.**
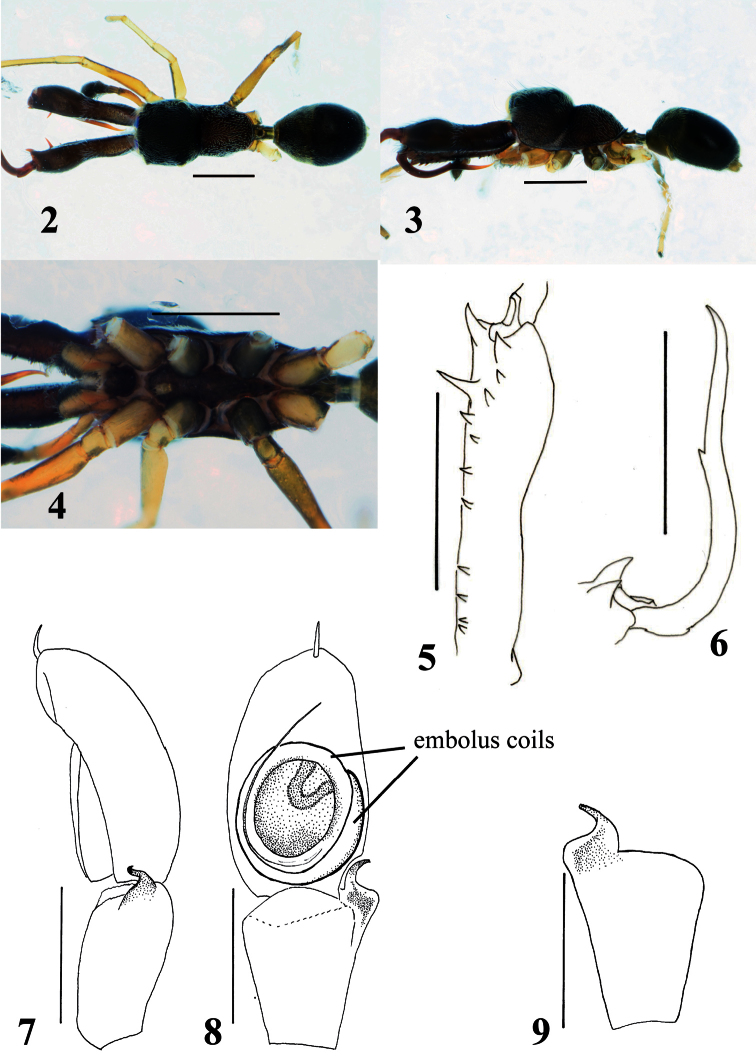
*Myrmarachne acutidens*, male. **2** Body in dorsal view **3** body in lateral view **4** endites, labium, coxae and trochanters in ventral view **5** left chelicera in ventral view **6** left fang in retrolateral view **7** left palp in retrolateral view **8** left palp in ventral view **9** left palpal tibia in dorsal view. (Scales. Figs **2–6**: 1 mm; **7–9**: 0.25 mm)

Blackish species; total length approximately 3.0–4.0 mm in males and 3.5–5.1 mm in females. Males distinguished from other species by structure of chelicera, which is anteriorly swollen and posteriorly narrow (Fig. 2); further distinguished from other species by first and second apical prolateral teeth of chelicera, which are long compared to other teeth (Fig. 5), and fang bearing short tooth-like apophysis submedially on its venter and weak protuberance on dorsum at its base (Fig. 6). Females distinguished from other species except *Myrmarachne macrognatha* (Thorell, 1894) by shape of dorsum of cephalic and thoracic parts, which are roundly convex (Fig. 11); *Myrmarachne acutidens* distinguished from *Myrmarachne macrognatha* by shape of proximal part of sclerotized copulatory ducts of epigyne, which is narrower in *Myrmarachne acutidens* (Figs 13 vs. 44).

#### Measurements

(male/female). Carapace length 1.50–2.00 (1.90) /1.88–2.20, width 0.83–1.17 (1.10) /0.88–1.07. Chelicera length 1.08– (1.83). ALE–PLE 0.58–0.77 (0.75) /0.65–0.75; ALE–PME 0.26– (0.35) /0.30–0.37. Width of eye row I 0.78–1.05 (1.02) /0.86–1.03; II 0.73–0.98 (0.95) /0.80–0.93; III 0.84–1.13 (1.08) /0.93–1.10. Eye size: AME 0.25–0.37 (0.33) /0.28–0.33; ALE 0.13–0.16 (0.15) /0.13–0.17; PME (0.05) /0.05; PLE 0.14–0.19 (0.16) /0.14–0.16.

**Male** (Figs 2–9). Cephalic part almost flat dorsally, distinctly higher than thoracic part; lateral surface of carapace strongly incised behind PLE; dorsum of thoracic part weakly convex in middle part, and then sloping downward (Figs 2–3). Chelicera almost as long as carapace, its anterior part weakly swollen dorsally and wider than posterior part; venter of chelicera bearing seven to ten prolateral and two to four retrolateral teeth; apical two prolateral and one retrolateral teeth large, especially second prolateral tooth counted from apex very long (Fig. 5). Fang weakly sinuous, bearing short tooth-like apophysis on venter near its middle, and weak protuberance on dorsum of its base (Fig. 6); proximal part of fang strongly arched. Pedicel short (0.25–0.32 mm). Abdomen oval without distinct constriction, and with two contiguous dorsal scuta; each lateral margin of where scuta are contiguous strongly incised at anterior third.

With palp in dorsal and ventral views, cymbium elongate-oval, with one apical spine (Fig. 8). Tegulum oval, with ejaculatory duct along proximal and prolateral margins, and rounded V-shaped ejaculatory duct at distal retrolateral margin of tegulum (Fig. 8). Embolus forming two oval coils; embolus coils occupying more than half of venter of cymbium, and basal coil as wide as venter of cymbium, while more ventral coil is slightly smaller (Fig. 8). With palp in dorsal and ventral views, RTA curved outward, with well-developed its flange; with palp in retrolateral view, RTA strongly s-curved with tip slightly spiraled (Figs 7, 9).

Number of spines on legs. Femur I pd 0–1, rd 0; tibia I pv 1–3, rv 2–3; metatarsus I pv 2, rv 2; tibia II pv 0, rv 0–2; metatarsus II pv 1, rv 1; femur III pd 1, rd 0; femur IV pd 1, rd 0–1.

Coloration in alcohol and pilosity. Carapace covered with white setae; cephalic part black and thoracic part dark brown; lateral surface of carapace above coxae I and II densely fringed with white setae (Figs 2–3). Chelicera dark brown; boundary between anterior swollen part and posterior narrow part fringed with white setae; anterior swollen part covered with long setae. Endite brownish cream. Labium and sternum dark brown (Fig. 4). Coxa and trochanter I dark yellow suffused with gray, coalesced into lateral dark stripe; coxae II and III black, distal prolateral part of coxa II yellowish suffused with gray, trochanter II like trochanter I except lighter yellow, trochanter III with more extensive lateral stripe and yellowish venter suffused with gray; coxa IV dark yellow with gray lateral stripes, trochanter IV white with short lateral gray marks (Fig. 4). Abdomen and its dorsal scuta black, covered with fine setae (Figs 2–3).

**Figures 10–14.**
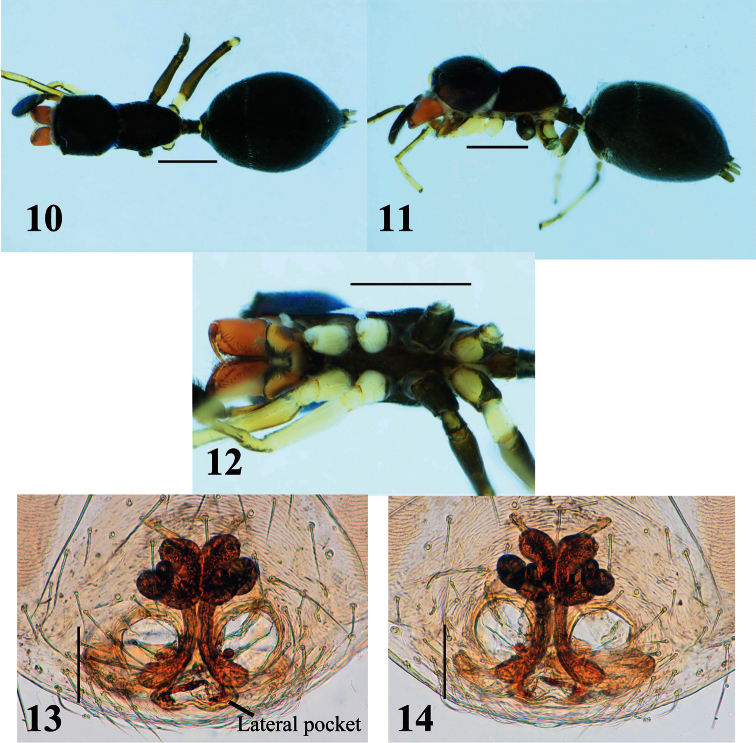
*Myrmarachne acutidens*, female. **10** Body in dorsal view **11** body in lateral view **12** endites, labium, coxae and trochanters in ventral view **13** internal structure of epigyne in ventral view **14** internal structure of epigyne in dorsal view. (Scales. Figs **10–12**: 1 mm; **13–14**: 0.1 mm)

**Figures 15–19.**
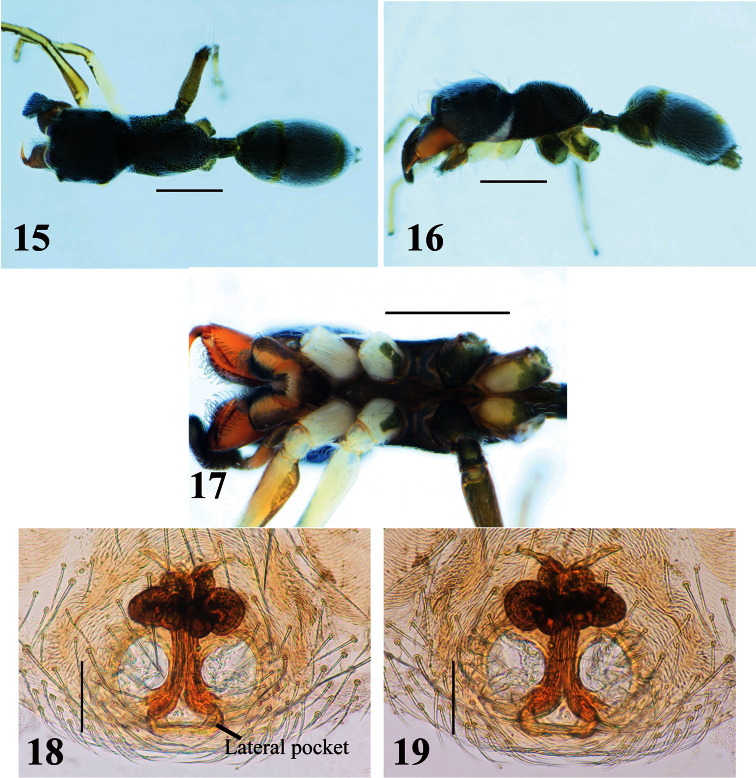
*Myrmarachne acutidens*, hairy female. **15** Body in dorsal view **16** body in lateral view **17** endites, labium, coxae and trochanters in ventral view **18** internal structure of epigyne in ventral view **19** internal structure of epigyne in dorsal view. (Scales. Figs **15–17**: 1 mm; **18–19**: 0.1 mm)

**Female** (Figs 10–19). With carapace in lateral view, cephalic part roundly convex dorsally, slightly higher than thoracic part; dorsal concavity behind PLE distinct; dorsum of thoracic part roundly convex overall (Figs 10–11, 15–16). Chelicera bearing four to six prolateral and seven to eight retrolateral teeth on its venter. Pedicel usually short, but sometimes long (0.23–0.95 mm). Abdomen oval, without distinct constriction (Figs 10–11, 15–16).

Epigyne (Figs 13–14, 18–19). Copulatory atria containing openings round. Median copulatory structure in front of epigastric furrow divided into lateral pockets; each lateral margin anteroposteriorly flattened. Sclerotized copulatory ducts emerging from oval spermathecae with complex twists, then extending between atria to vicinity of lateral pockets.

Number of spines on legs. Tibia I pv 4–5, rv 4; metatarsus I pv 2, rv 2; tibia II pv 0–1, rv2; metatarsus II pv 1–2, rv 2.

Coloration in alcohol and pilosity. Carapace black; cephalic part covered with fine setae; thoracic part sparsely covered with white setae; lateral surface of carapace above coxae I and II densely fringed with white setae (Figs 10–11, 15–16). Chelicera light brown. Endite brownish yellow, tinged with gray (Figs 12, 17). Labium cream, tinged with black (Figs 12, 17). Sternum black (Figs 12, 17). Coxae and trochanters similar to male pattern but lighter in color; coxae and trochanters I and II white, coxa II with variable black retrolateral stripe; coxa and trochanter III black, trochanter lighter ventrally suffused with black; coxa IV yellow with extensive lateral black stripe, trochanter IV white (Figs 12, 17). Abdomen black, covered with fine setae; some white setae roughly forming transverse white band in anterior dorsum of abdomen (Figs 10–11, 15–16).

#### Etymology.

The specific name is derived from the second prolateral tooth counted from the apex in males.

#### Biology.

The species is arboreal, and collected from trees in secondary forests or plantation areas.

#### Distribution.

Flores.

### Myrmarachne epigealis sp. n.

urn:lsid:zoobank.org:act:F33ED9E3-EC1F-4211-96FA-86927ADF099E

http://species-id.net/wiki/Myrmarachne_epigealis

Figs 20 –32

#### Type material.

Holotype male (MZB. Aran. 505), Moni [=8°45'S, 121°51'E], Flores, East Nusa Tenggara Prov., INDONESIA, 18–19.X.2012, T. Yamasaki leg. Paratypes: 1 male (FSCA), Mego [=8°40'S, 122°2'E], Sikka, Flores, East Nusa Tenggara Prov., INDONESIA, 16.X.2012, T. Yamasaki leg.; 2 females (FSCA), same as holotype; 1 female (MZB. Aran. 506), same loc., 18.X.2012, Rijal Satria leg.

#### Diagnosis.

**Figures 20–27.**
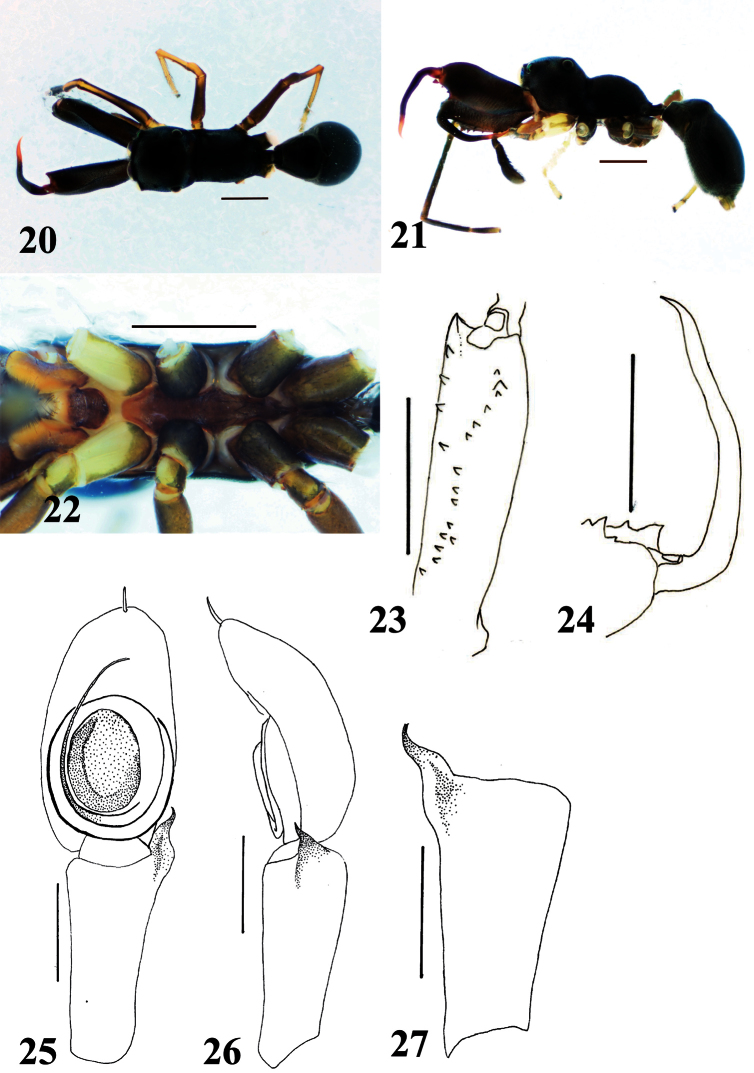
*Myrmarachne epigealis*, male. **20** Body in dorsal view **21** body in lateral view **22** endites, labium, coxae and trochanters in ventral view **23** left chelicera in ventral view **24** left fang in retrolateral view **25** left palp in ventral view **26** left palp in retrolateral view **27** left palpal tibia in dorsal view. (Scales. Figs **20–24**: 1 mm; **25–27**: 0.25 mm)

**Figures 28–32.**
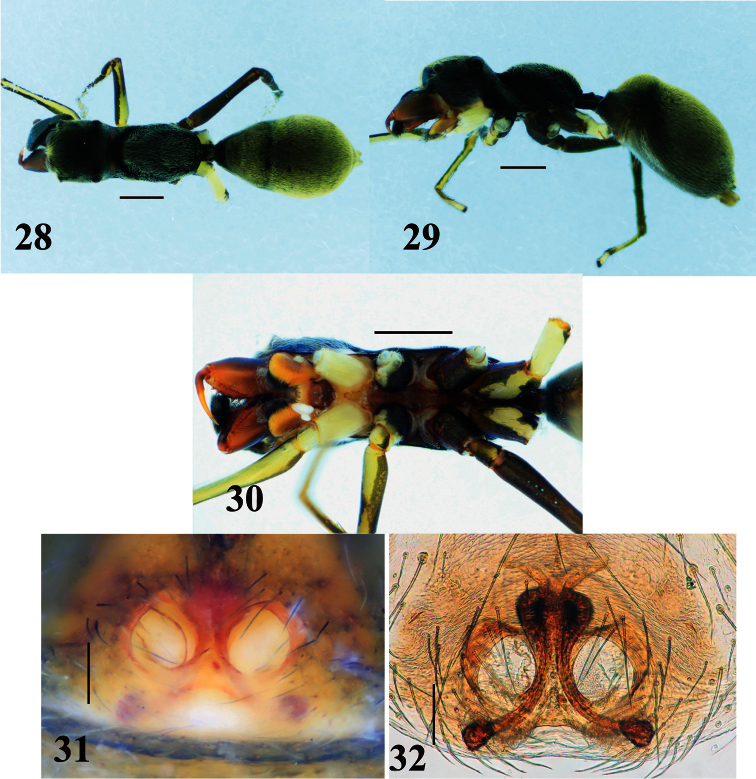
*Myrmarachne epigealis*, female. **28** Body in dorsal view **29** body in lateral view **30** endites, labium, coxae and trochanters in ventral view **31** epigyne in ventral view **32** internal structure of epigyne in ventral view. (Scales. Figs **28–30**: 1 mm; **31–32**: 0.1 mm)

Black species; total length approximately 5.3–5.8 mm in males, 6.2–7.2 mm in females. Males distinguished from other species by prolateral dorsal margin of chelicera diverted toward retrolateral side in distal half, exposing prolateral surface of chelicera in dorsal view (Fig. 20). Females distinguished from other species except *Myrmarachne grossa* Edmunds & Prószyński, 2003 by thoracic part, which is longer than cephalic part (Figs 28–29); *Myrmarachne epigealis* distinguished from *Myrmarachne grossa* by sclerotized copulatory ducts, which are narrower and longer than those of *Myrmarachne grossa* (cf. figs 78–79 in Edmunds and Prószyński 2003).

#### Measurements

(male/female). Carapace length 2.63– (2.83) / 3.20–3.35, width 1.35– (1.40) /1.33–1.48. Chelicera length 1.90– (2.07). ALE–PLE 0.95– (1.00) /1.03–1.13; ALE–PME 0.45– (0.47) / 0.50–0.55. Width of eye row I 1.28– (1.33) / 1.38–1.45; II 1.13– (1.18) /1.20–1.28; III 1.33– (1.38) / 1.43–1.53. Eye size: AME (0.43) / 0.43–0.45; ALE 0.22– (0.23) /0.22–0.23; PME 0.06– (0.08) /0.08; PLE 0.23– (0.24) / 0.25–0.26.

**Male** (Figs 20–27). Cephalic part weakly convex dorsally, distinctly higher than thoracic part; in lateral view, dorsum of carapace sloping downward behind PLE, and concave between cephalic and thoracic parts; lateral surface between cephalic and thoracic parts weakly incised; thoracic part roundly swollen overall (Figs 20–21). Chelicera slightly shorter than carapace; with chelicera in dorsal view, its dorsal prolateral margin diverted toward retrolateral margin in distal half, exposing prolateral surface (Fig. 20); venter of chelicera bearing four to five prolateral and eleven to 14 retrolateral teeth (Fig. 23); apical prolateral corner of venter forming large tooth-like apophysis; with chelicera in ventral view, one retrolateral tooth present next to third retrolateral tooth counted from base, and one prolateral tooth slightly proximal to row of retrolateral teeth. Fang weakly sinuous, without tooth-like apophysis (Fig. 24). Pedicel short (0.30–0.43 mm). Abdomen oval with weak constriction at anterior fourth, and its entire dorsum covered with two contiguous dorsal scuta; lateral margins strongly incised where scuta come together (Figs 20–21).

With palp in dorsal and ventral views, cymbium elongate-oval with one apical spine (Fig. 25). Tegulum oval, with long seminal reservoir along margin of tegulum (Fig. 25). Embolus forming two oval coils; more ventral embolus coil as wide as venter of cymbium, slightly narrower than basal coil (Fig. 25). RTA well developed with the tip curved inward, and without a distinct its flange (Figs 26–27).

Number of spines on legs. Patella I pv 0, rv 0–1; tibia I pv 5, rv 5; metatarsus I pv 2, rv 2; tibia II pv 3, rv 3; metatarsus II pv 2, rv 2.

Coloration in alcohol and pilosity. Carapace and chelicera black, covered with very short inconspicuous setae (Figs 20–21). Carapace with pair of light brown spots behind PLE. Endite, labium and sternum light brown, tinged with black (Fig. 22). In ventral view, coxa and trochanter I light yellow, tinged with gray on posterior margin of coxa and laterally on trochanter; coxa II black, trochanter II like trochanter I; coxa and trochanter III black, trochanter yellow ventrally strongly tinged with black; coxa IV blackish with yellow venter strongly tinged with black, trochanter IV light yellow (Fig. 22). Abdomen and its dorsal scuta black, covered with fine setae (Figs 20–21).

**Female** (Figs 28–32). Cephalic part very weakly convex or almost flat, higher than thoracic part; with carapace in lateral view, dorsal concavity roundly convex behind PLE; thoracic part swollen dorsally overall (Figs 28–29). Chelicera bearing seven prolateral and 13 to 14 retrolateral teeth on its venter. Pedicel short (0.43–0.55 mm). Abdomen oval with weak constriction in anterior third, and without obvious dorsal scuta (may be present under pilosity).

Epigyne (Figs 31–32). Copulatory atria containing openings round. Median pocket present in front of epigastric furrow, weakly sclerotized. Sclerotized copulatory ducts extending from oval spermathecae without twists to posterolateral to atria.

Number of spines on legs. Patella I pv 0, rv 1; tibia I pv 5, rv 5; metatarsus I pv 2, rv 2; tibia II pv 3, rv 3; metatarsus II pv 2, rv 2.

Coloration in alcohol and pilosity. Carapace mainly black, covered with white setae densely; carapace with pair of light brown spots behind PLE; lateral surface of carapace above coxa I white, densely fringed with white setae (Figs 28–29). Chelicera brown. Endite, labium and sternum brownish cream, weakly tinged with black (Fig. 30). Coxae and trochanters in ventral view similar to those of males, except coxa IV with much more yellow ventrally, and trochanter IV yellow with distal retrolateral black spot (Fig. 30). Abdomen covered with golden setae dorsally, and with fine setae ventrally (Fig. 28).

#### Etymology.

The specific name is derived from the ground microhabitat, where the species often occurs.

#### Biology.

The species occurs in lower vegetation and on the ground.

#### Distribution.

Flores.

### Myrmarachne macrognatha

(Thorell, 1894)

http://species-id.net/wiki/Myrmarachne_macrognatha

Figs 33 –45

Salticus macrognathus Thorell, 1894: 58.
Myrmarachne macrognatha : Roewer, 1954: 947.

#### Type material.

Holotype male (NRM), Java, [INDONESIA], van Hass. [van Hasselt].

**Non-type material examined**: 16 males and 11 females, Moni [=8°45'S, 121°51'E], Flores, East Nusa Tenggara Prov., INDONESIA, 17–19.X.2012, T. Yamasaki leg.

#### Diagnosis.

**Figures 33–40.**
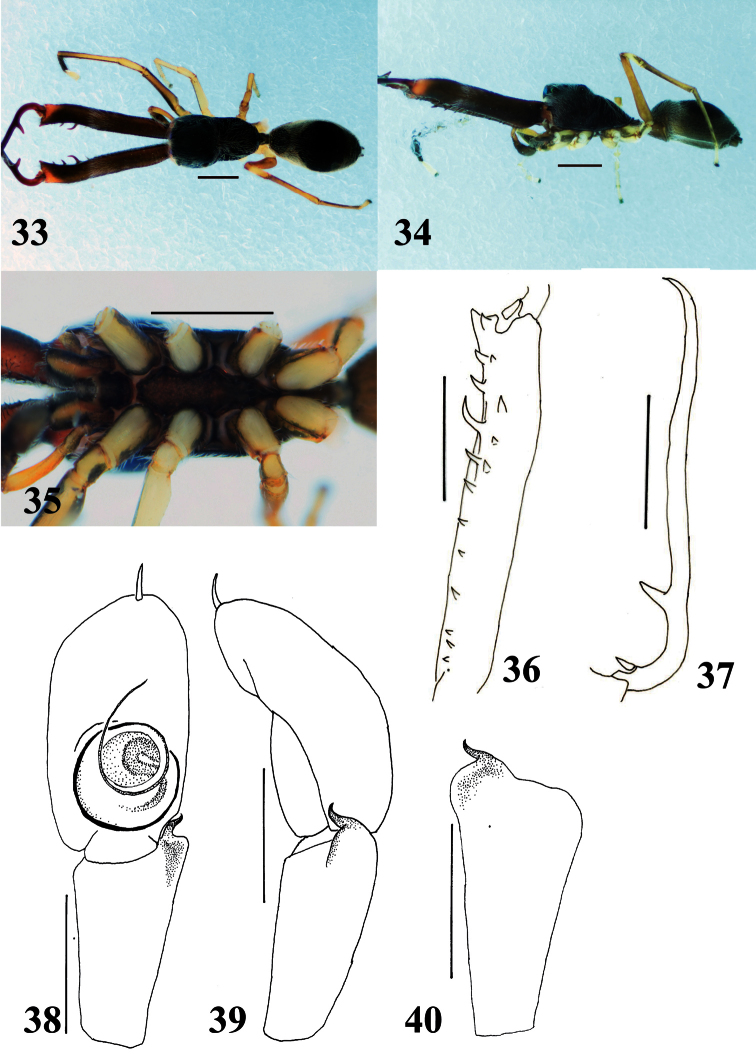
*Myrmarachne macrognatha*, male. **33** Body in dorsal view **34** body in lateral view **35** endites, labium, coxae and trochanters in ventral view **36** left chelicera in ventral view **37** left fang in retrolateral view **38** left palp in ventral view **39** left palp in retrolateral view **40** left palpal tibia in dorsal view. (Scales. Figs **33–37**: 1 mm; **38–40**: 0.25 mm).

**Figures 41–45.**
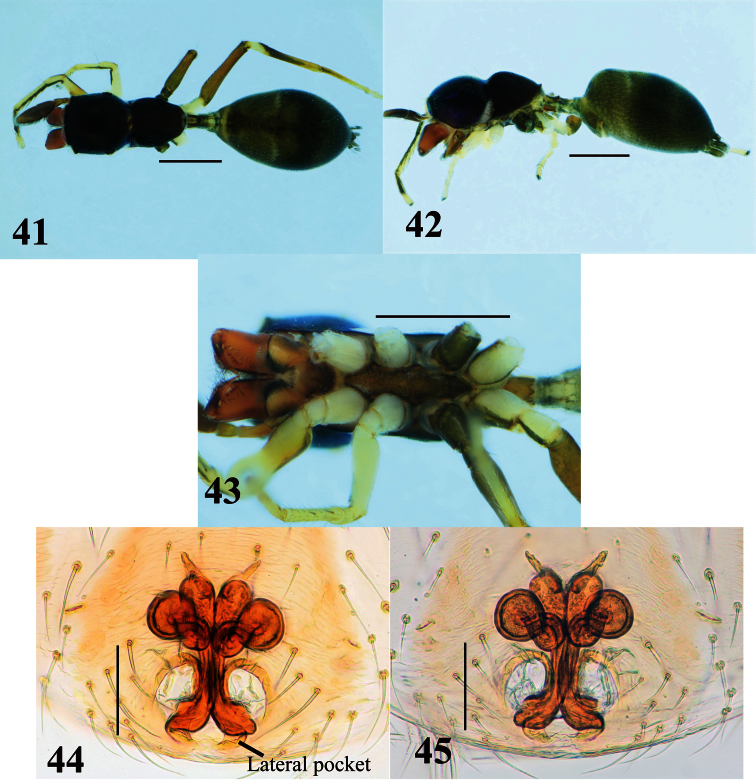
*Myrmarachne macrognatha*, female. **41** Body in dorsal view **42** body in lateral view **43** endites, labium, coxae and trochanters in ventral view **44** internal structure of epigyne in ventral view **45** internal structure of epigyne in dorsal view. (Scales. Figs **41–43**: 1 mm; **44–45**: 0.1 mm)

Blackish species; total length approximately 3.2–5.3 mm in males and 4.0–5.2 mm in females. Males distinguished from other species except *Myrmarachne smaragdina* Ceccarelli, 2010 (cf. fig. 34 in Ceccarelli 2010) by characteristic dentition of chelicera, of which fourth prolateral tooth counted from apex long (Fig. 36); further distinguished from *Myrmarachne smaragdina* by shapes of chelicera and carapace (figs 28–29 in Ceccarelli 2010 vs. Figs 33–34). Females distinguished from other species except *Myrmarachne melanocephala* MacLeay, 1839 by sclerotized copulatory ducts clearly twisted in “figure 8” (Figs 44–45); *Myrmarachne macrognatha* distinguished from *Myrmarachne melanocephala* by absence of distinct markings on abdomen (Figs 41 vs. 54).

#### Measurements (male/female).

Carapace length 1.63–2.50 (2.23) /1.75–2.10, width 0.93–1.57 (1.38) /0.91–1.08. Chelicera length 1.30–3.85 (3.30). ALE–PLE 0.67–1.02 (0.92) /0.68–0.82; ALE–PME 0.30–0.43 (0.42) /0.26–0.35. Width of eye row I 0.87–1.30 (1.17) /0.93–1.05; II 0.80–1.20 (1.10) /0.85–0.95; III 0.95–1.45  (1.32) /1.02–1.15. Eye size: AME 0.28–0.42 (0.37) /0.31–0.34; ALE 0.15–0.20 (0.18) /0.14–0.16; PME 0.05– (0.08) /0.05–0.06; PLE 0.15–0.23 (0.20) /0.16–0.18.

**Male** (Figs 33–40). With carapace in lateral view, cephalic part almost flat dorsally, higher than thoracic part; dorsal concavity behind PLE shallow or indistinct; thoracic part sloping downward, not distinctly convex dorsally (Figs 33–34). Chelicera distinctly longer than carapace, each lateral margin almost parallel-sided except for distinctly convex prolateral margin near anterior end of chelicera; venter of chelicera bearing nine to twelve prolateral and three to six retrolateral teeth; fourth prolateral tooth counted from apex long and strongly curved (Fig. 36). Fang almost straight or very weakly sinuous except for curved tip and base, with long tooth-like apophysis on its venter at about 1/4 the length of fang from its base (Fig. 37). Pedicel short (0.18–0.30 mm). Abdomen oval without distinct constriction, with dorsal two scuta that are clearly separated.

With palp in dorsal and ventral views, cymbium elongate-oval, with one apical spine (Fig. 38). Tegulum round and small, with s-curved ejaculation duct in distal retrolateral part of tegulum (Fig. 38). Embolus forming two round coils; embolus coils occupying less than half of venter of cymbium; ventral coil much smaller than basal coil, only about half the diameter (Fig. 38). RTA strongly curved, and in retrolateral view, somewhat spiraled (Fig. 38–40). Flange of RTA moderately developed.

Number of spines on legs. Femur I pd 1, rd 0; tibia I pv 0–3, rv 1–4; metatarsus I pv 2, rv 2; femur II pd 1, rd 0; tibia II pv 0, rv 0–2; metatarsus II pv 0–2, rv 1–2; femur III pd 0–1, rd 0; femur IV pd 1, rd 0.

Coloration in alcohol and pilosity. Carapace black; cephalic part covered with fine white setae, and thoracic part sparsely covered with white setae; lateral carapace not fringed with white setae (Figs 33–34). Chelicera dark brown to black, and long white setae roughly forming transverse band in anterior part of chelicera. Endite and labium brownish orange, tinged with black laterally (Fig. 35). Coxae and trochanters I, III, IV yellow with gray lateral stripes; coxa and trochanter II cream-white, coxa with gray lateral stripes; in large specimens, coxae sometimes strongly tinged with black (Fig. 35). Abdomen and its dorsal scuta black, covered with white long setae and fine setae dorsally (Figs 33–34).

**Female** (Figs 41–45). With carapace in lateral view, cephalic part weakly convex dorsally, slightly higher than thoracic part; dorsal concavity behind PLE distinct; thoracic part swollen dorsally (Figs 41–42). Chelicera bearing five to six prolateral and six retrolateral teeth on its venter. Pedicel relatively longer than that of males (0.35–0.43 mm). Abdomen oval, without distinct constriction and dorsal scutum.

Epigyne (Figs 44–45). Copulatory atria containing openings oval. Lateral pockets present in front of epigastric furrow, anteroposteriorlly flattened. Sclerotized copulatory ducts clearly twisted in “figure 8” adjacent to cylindal spermathecae, then extending between atria to approximately the area of the lateral pockets.

Number of spines on legs. Tibia I pv 3–4, rv 3–4; metatarsus I pv 2, rv 2; tibia II pv 0, rv 2; metatarsus II pv 0–2, rv 2.

Coloration in alcohol and pilosity. Carapace dark brown to black; cephalic part covered with white and fine setae; thoracic part covered with white setae; lateral surface of carapace above coxae I and II densely fringed with white setae; white setae roughly forming white diagonal band from above coxa IV to upper dorsum (Figs 41–42). Chelicera dark brown. Endite, labium and sternum brownish orange, weakly tinged with black. Coxae and trochanters I and II white; coxa and trochanter III black, trochanter with yellow venter; coxa and trochanter IV pale yellow with lateral gray stripes (Fig. 43). Abdomen gray, covered with fine setae; two spots behind two white partial transverse bands present dorsally in anterior part of abdomen (Figs 41–42).

#### Remarks.

The specimens from Flores are slightly different from the holotype of *Myrmarachne macrognatha* from Java in the cheliceral teeth. The third prolateral tooth counted from the apex of the Flores specimens is shorter than that of the holotype (which has both the third and fourth teeth elongate). Although the number and size of cheliceral teeth is variable, generally corresponding to body size, the short apical third prolateral tooth seems to be a stable character within our specimens collected from Flores regardless of body size. However, we regard the Flores specimens as *Myrmarachne macrognatha* on the basis of many other morphological similarities. To understand geographical variation of the species, a phylogenetic study based on molecular analysis is needed in the future.

#### Biology.

*Myrmarachne macrognatha* is an arboreal species, and very common and abundant in eastern Flores.

#### Distribution.

Java, Flores.

### Myrmarachne melanocephala

MacLeay, 1839

http://species-id.net/wiki/Myrmarachne_melanocephala

Figs 46 –58

Myrmarachne melanocephala MacLeay, 1839: 11, pl. 1, fig. 4; Galiano 1969: 146; Edwards and Benjamin 2009: 5, figs. 1A–H, 2A–D, 3A–D, 4A–E, 5A–D.
Mymecia melanocephala Walckenaer, 1841: 462.
Salticus contractus Karsch, 1880: 396.
Salticus providens Peckham & Peckham, 1892: 34.
Myrmarachne providens : Simon 1901: 500.
Myrmarachne ramosa Badcock, 1918: 303, fig. 8; Edmunds and Prószyński 2003: 301, figs. 8–21.
Myrmarachne albicrurata Badcock, 1918: 306, fig. 9a.
Myrmarachne lateralis Badcock, 1918: 310, figs. 9b–c.

#### Non-type material examined.

1 male and 2 females. Mego [=8°40’S, 122°2’E], Sikka, Flores, East Nusa Tenggara Prov., INDONESIA, 16.X.2012, T. Yamasaki leg.

#### Diagnosis.

**Figures 46–53.**
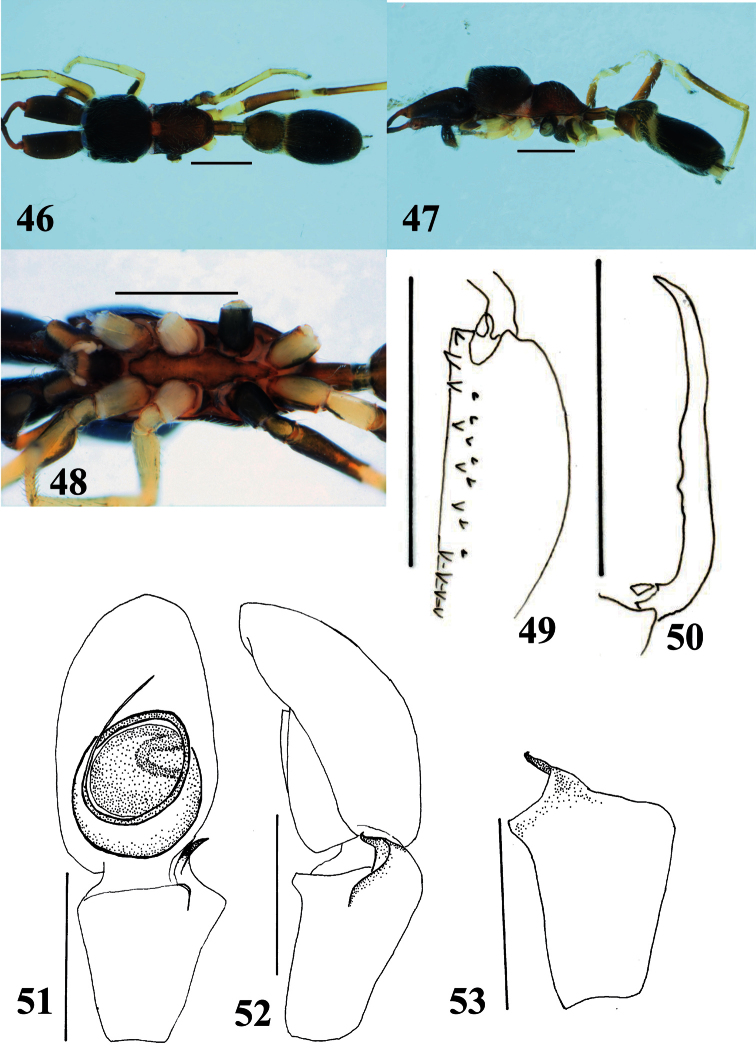
*Myrmarachne melanocephala*, male. **46** Body in dorsal view **47** body in lateral view **48** endites, labium, coxae and trochanters in ventral view **49** left chelicera in ventral view **50** left fang in retrolateral view **51** left palp in ventral view **52** left palp in retrolateral view **53** left palpal tibia in dorsal view. (Scales. Figs **46–50**: 1 mm; **51–53**: 0.25 mm).

**Figures 54–58.**
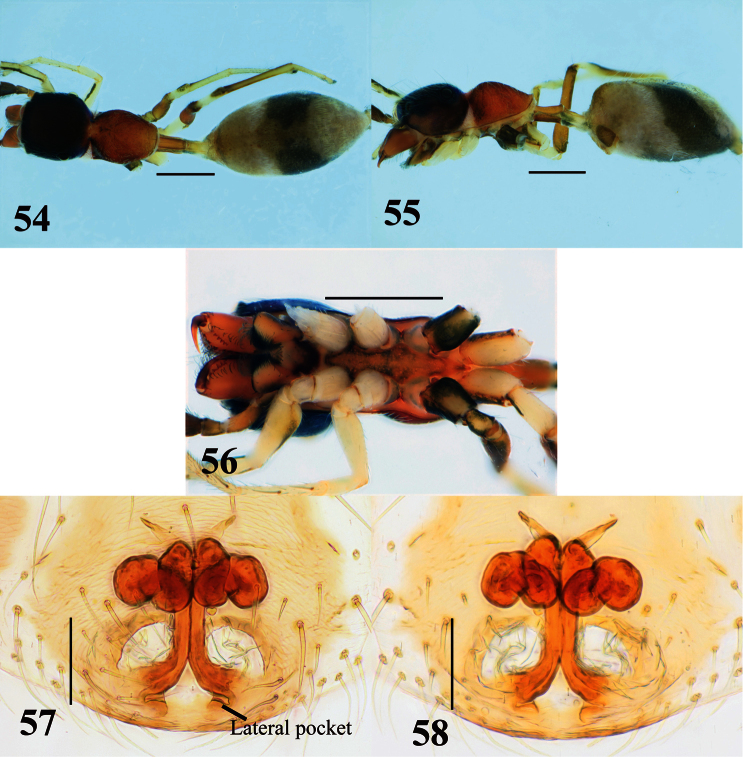
*Myrmarachne melanocephala*, female. **54** Body in dorsal view **55** body in lateral view **56** endites, labium, coxae and trochanters in ventral view **57** internal structure of epigyne in ventral view **58** internal structure of epigyne in dorsal view. (Scales. Figs **54–56**: 1 mm; **57–58**: 0.1 mm)

Slender, light to dark brown species with relatively long pedicel. Males distinguished from other species by pedicel, which is as long as ALE–PLE (Fig. 46); further distinguished from species having long pedicel such as *Myrmarachne assimilis* Banks, 1930 (cf. figs 7, 13 in Banks 1930), *Myrmarachne cornuta* Badcock, 1918 (cf. figs 30–35 in Edmunds and Prószyński 2003) and *Myrmarachne plataleoides* (O. Pickard-Cambridge, 1869) (cf. figs 1–7 in Edmunds and Prószyński 2003) by shape and dentition of chelicera (Fig. 49). Females distinguished from others species by long pedicel as in males (Fig. 54); further distinguished from species having long pedicel such as *Myrmarachne assimilis*, *Myrmarachne cornuta*, *Myrmarachne glavisi* Prószyński & Deeleman-Reinhold, 2010 and *Myrmarachne plataleoides* by markings on abdomen (Fig. 54) and structure of epigyne (Figs 57–58).

#### Measurements (male/female).

Carapace length 2.13/2.18–2.37, width 1.15/1.10–1.18. Chelicera length 1.22. ALE–PLE 0.82/0.77–0.83; ALE–PME 0.40/0.36–0.38. Width of eye row I 1.03/1.03–1.12; II 0.97/1.00–1.08; III 1.13/1.15–1.20. Eye size: AME 0.33/0.33–0.35; ALE 0.18/0.16–0.17; PME 0.06/0.05; PLE 0.19/0.19.

**Male** (Figs 46–53). Cephalic part almost flat dorsally, higher than thoracic part; thoracic part swollen dorsally; strongly incised laterally between cephalic and thoracic part (Figs 46–47). Chelicera shorter than carapace, bearing eleven prolateral and seven retrolateral teeth on its venter; fang weakly sinuous, without distinct tooth-like apophysis (Figs 49–50). Pedicel relatively long (0.50 mm). Abdomen elongate-oval; two dorsal scuta strongly incised laterally between them (Figs 46–47).

With palp in dorsal and ventral views, cymbium oval, without distinct spines on its apex (Fig. 51). Tegulum round, with C-shaped ejaculatory duct in its distal retrolateral part (Fig. 51). Embolus forming two round coils; ventral coil much narrower than basal coil, and basal coil slightly narrower than venter of cymbium (Fig. 51). RTA strongly curved and tip spiraled, with well-developed flange (Figs 52–53).

Number of spines on legs. Femur I pd 1, rd 0; tibia I pv 4, rv 4; metatarsus I pv 2, rv 2; tibia II pv 0, rv 2; metatarsus II pv 2, rv 2.

Coloration in alcohol and pilosity. Cephalic part dark brown, covered with white setae; thoracic part light brown, sparsely covered with white setae; lateral surface above coxa II densely fringed with white setae (Figs 46–47). Chelicera dark brown. Endite and labium brownish orange, tinged with black, especially labium (Fig. 48). Sternum brownish orange (Fig. 48). Coxae and trochanters I, II and IV white, IV with lateral black stripes; coxa and trochanter III black except venter of trochanter light brown (Fig. 48). Abdomen covered with fine setae; anterior dorsal scutum grayish pale brown, and posterior dorsal scutum black; white transeverse band between scuta running diagonally from lateral to ventral area (Figs 46–47).

**Female** (Figs 54–58). Carapace strongly incised laterally behind PLE; cephalic part almost flat dorsally, slightly higher than thoracic part; thoracic part swollen dorsally (Figs 54–55). Chelicera bearing five prolateral and six retrolateral teeth on its venter. Pedicel relatively long (0. 58–0.83 mm). Abdomen elongate-oval, dorsal scuta inconspicuous (Figs 54–55).

Epigyne (Figs 57–58). Copulatory atria laterally-oriented oval. Lateral pockets present in front of epigastric furrow, rather widely separated. Sclerotized copulatory ducts from “figure 8” adjacent to ovoid spermathecae, then extending between atria to approximate vicinity of widely separated lateral pockets.

Number of spines on legs. Tibia I pv 4, rv 4–5; metatarsus I pv 2, rv 2; tibia II pv 1–3, rv 2–3; metatarsus II pv 2, pv 2.

Coloration in alcohol and pilosity. Cephalic part black, covered with sparse white setae dorsally; thoracic part light to dark brown, sparsely covered with white setae; lateral surface of carapace above coxa II white, densely fringed with white setae (Figs 54–55). Chelicera light brown. Endite, labium and sternum brownish orange, weakly tinged with black laterally (Fig. 56). Coxae and trochanters similar to those of males, except venter of coxa III also light brown (Fig. 56). Abdomen covered with white and fine setae (Figs 54–55).

#### Remarks.

*Myrmarachne melanocephala* are very similar to *Myrmarachne glavisi*, a long-pedicled species, in males (cf. figs 117, 119–120 in Prószyński and Deeleman-Reinhold 2010). However, the females of *Myrmarachne melanocephala* are distinguishable from the females of *Myrmarachne glavisi* by the structure of the epigyne (cf. figs 125–126 in Prószyński and Deeleman-Reinhold 2010). We here consider *Myrmarachne melanocephala* different from *Myrmarachne glavisi*. However, the taxonomic status of *Myrmarachne glavisi* should be reviewed by a detailed comparison between the type materials of *Myrmarachne glavisi* and *Myrmarachne melanocephala* in the future.

The male specimen examined in the present study has relatively short chelicera compared with specimens from other areas. The male chelicera varies in the length depending on the body size within a species. The female specimens examined here agree with the description of *Myrmarachne melanocephala* and specimens from other areas, in particular the gray abdomen with transverse median black band is typical of populations throughout Southeast Asia, but lighter in color than the mostly black topotypical specimens from India. This seems to be only regional color variation.

#### Biology.

The specimens were collected from plantation areas.

#### Distribution.

Widely distributed in South and Southeast Asia.

## Supplementary Material

XML Treatment for Myrmarachne acutidens

XML Treatment for Myrmarachne epigealis

XML Treatment for Myrmarachne macrognatha

XML Treatment for Myrmarachne melanocephala
